# Antibody–Drug Conjugates: A Start of a New Era in Gynecological Cancers

**DOI:** 10.3390/curroncol31110522

**Published:** 2024-11-13

**Authors:** Samir Fasih, Stephen Welch, Ana Elisa Lohmann

**Affiliations:** 1 Department of Oncology, Division of Medical Oncology, University of Western Ontario, London, ON N6A 5W9, Canada; samir.fasih@lhsc.on.ca (S.F.); stephen.welch@lhsc.on.ca (S.W.); 2 Department of Epidemiology and Biostatistics, University of Western Ontario, London, ON N6A 5W9, Canada

**Keywords:** antibody drug conjugates, gynecological cancers, ovarian cancer, cervical cancer

## Abstract

Antibody–drug conjugates (ADCs) are a new class of therapeutic agents designed to target specific antigens on tumor cells, combining the specificity of monoclonal antibodies with the cytotoxicity of chemotherapy agents. ADCs have been available for over a decade, but in gynecological cancers, these agents are relatively new with great promise ahead. More than 80% of ongoing trials in gynecological cancers are evaluating ADCs’ safety and efficacy, of which 40% are early-phase trials. Around twenty ADCs are currently under investigation, either alone or in combination with chemotherapies or immune checkpoint inhibitors. Among them, mirvetuximab soravtansine has been recently approved by the Food and Drug Administration (FDA) in platinum-resistant ovarian cancer with high folate-α receptor expression, as a single agent or in combination. Tisotumab vedotin and trastuzumab deruxtecan are also now approved by the FDA in patients with pre-treated cervical and uterine cancers and further investigation is ongoing. Overall, the toxicity profiles of ADCs are acceptable. Ocular toxicity is one of the specific side effects of some ADCs, but most of the cases are manageable with the use of prophylactic steroids and dose adjustments. This review aims to provide an overview of the fundamental and operational features of ADCs and examine the latest and most promising data, with a particular focus on the Canadian viewpoint.

## 1. Introduction

Gynecological cancers are a group of neoplasms of female reproductive organs and genitals, including carcinomas of the vagina, vulva, cervix, uterus, ovaries, and fallopian tubes. Based on the statistics latest data available until the year 2022, cancer is the most common cause of death in Canada [1]. The most common gynecological cancer is uterine cancer (7.4%), followed by ovarian (2.7%) and cervical (1.4%), respectively [2]. The treatment of gynecological tumors depends on the cancer type and disease stage. Patients with distant metastasis or recurrences have a poor outcome with uterine and cervical cancer, having a 5-year survival rate of around 18% [3]. In ovarian cancer, the majority of patients present with advanced disease at initial diagnosis [4]. Despite excellent responses to surgery and chemotherapy, more than 80% [5] will relapse and eventually become platinum resistant. Recently, many immunotherapeutic treatments, including immune checkpoint inhibitors and monoclonal antibodies, have been used for treatment in gynecological malignancies, especially for uterine and cervical carcinomas, improving the therapeutic options of these diseases [6,7,8]. Antibody–drug conjugates (ADCs) have been in use for more than a decade in other cancers but in gynecological cancers they have recently come up, with a great promise for the future.

The first antibody–drug conjugate (ADC) utilized in clinical settings was gemtuzumab ozogamicin for acute myeloid leukemia, approved by the Food and Drug Administration (FDA) in 2001 [9]. Following that, brentuximab vedotin received approval for Hodgkin lymphoma [10]. Trastuzumab emtansine (T-DM1) was the first ADC approved by the FDA for solid tumors, used in the treatment of metastatic as well as early-stage breast cancer [11,12]. These ADCs feature a unique structure and design, employing monoclonal antibodies to selectively deliver potent cytotoxic agents directly to the tumor site. Theoretically, this design aims to target only the cancer cells while preserving healthy tissues. In the early 20th century, Paul Ehrlich coined the concept of “magic bullets”, suggesting that certain compounds could reach specific cellular targets to treat diseases [13]. Since then, the field has advanced rapidly, with more than 100 different types of ADCs currently being investigated for use in both solid and hematological malignancies. To date, sixteen ADCs have been approved by the FDA, the European Medicines Agency (EMA), and other regulatory bodies, and have been launched in the market for the treatment of hematologic malignancies and solid tumors [14].

In this article, we discuss the use of new antibody drug conjugates for treating patients with various gynecologic cancers.

## 2. ADCs: Mechanism of Action

### 2.1. Structure

**Figure 1:**
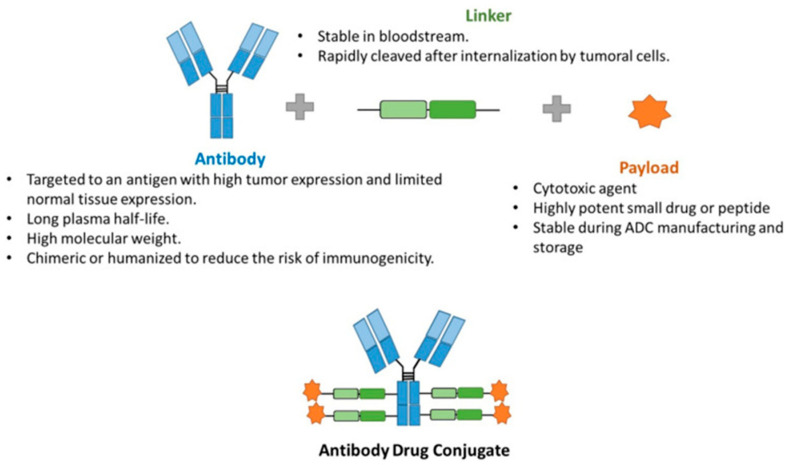
Scheme of the general structure of an ADC [12].

ADCs consist of three structural components that play a crucial role in determining the effectiveness and safety of the drug. The ADCs consist of a targeted antibody which is attached to a potent cytotoxic agent called the ‘payload’ via a chemical ‘linker’ [11]. The three structural components with their characteristics are shown in Figure 1 [12].

#### 2.1.1. Antibody

The first component is a highly selective monoclonal antibody that specifically targets a tumor-associated antigen, with minimal expression on other tissues [13]. The latest ADCs are developed from fully humanized antibodies, typically of the IgG type. The utilization of fully humanized antibodies has been instrumental in reducing the immunogenicity of earlier ADCs, which were created using murine and chimeric components [14]. IgG1 antibodies are now more commonly used because of their overall stability in systemic circulation, with a long half-life of 2 to 3 weeks and a strong influence on innate immune cells, such as natural killer (NK) cells and macrophages, through interactions with Fcγ receptors [15].

#### 2.1.2. Payload

The second component is the drug conjugate, also known as the payload, which typically consists of a conventional chemotherapy agent. Payloads refer to cytotoxic molecules that are very small and typically measure in the nanomolar or picomolar range. These molecules are attached to the structure of an antibody through a linker. Throughout the years, various classes of payloads have been created, with auristatins and maytansinoids being the most commonly used [16]. Auristatins are man-made compounds that mimic dolostatin, a natural substance that inhibits the assembly of tubulin [17]. The most commonly used auristatin is monomethyl auristatine E (MMAE), also known as vedotin. Another group of frequently used compounds are maytansinoids, which are synthetic versions of maytansine and function similarly to vinca alkaloids by inhibiting microtubule assembly. Maytansinoids, such as mertansine (DM1), emtansine, soravtansine, and ravtansine (DM4), are commonly used [18]. Calicheamicin, duocarmicins, and pyrrolobenzodiazepines are examples of additional payloads that have been developed for use in clinical practice. These payloads possess potent inhibitory properties against nucleic acid synthesis due to their unique capability to identify and attach to specific sequences within the minor groove of DNA. More recently, Topoisomerase I (TOP1) inhibitors constitute an emerging payload class to engineer antibody drug conjugates labelled as next-generation ADCs. These Exatecan-based linker–payload complexes are more potent and stable and can carry a higher concentration of drug to antibody ratio (DAR). One other factor is that hydrophobicity can determine the efficacy and toxicity of an ADC. Hydrophobic payloads can diffuse from the target expressing cells to adjacent normal cells, a phenomenon called the “bystander effect”. This occurrence is very important, especially in regards to the heterogeneity of tumors, as the therapeutic effects are enhanced due to the bystander effect [19,20]. At the same time, the level of hydrophobicity can affect the penetration of the payload into the liver, causing liver toxicity if it is less hydrophobic, or the payload can be taken up by tissues, causing hematological and ocular toxicities [21,22,23]. The fine-tuning of payloads is necessary to maintain the bystander effect yet also maintain the efficacy by controlling the drug to antibody ratio (DAR) [24]. Other than the conventional payloads, immunomodulators [25] and protein-degrader-recruiting molecules [26] have recently emerged as novel payloads.

#### 2.1.3. Linker

The final component is the linker, which joins the monoclonal antibody to the drug conjugate. It maintains the stability of the drug conjugate in the bloodstream while effectively releasing the payload inside the target cell [27]. Linkers are categorized into two types: cleavable and non-cleavable [27]. Cleavable linkers can be broken down by reactions involving proteases, acidic pH, endosomes, or lysosomes. This allows some of the cytotoxic payload to be released into the tumor microenvironment, impacting both antigen-expressing target cells and nearby non-antigen-expressing cells through the bystander effect [28]. Conversely, non-cleavable linkers rely on the lysosomal proteolytic activity of the antibody to release the payload. When non-cleavable linkers are cleaved, the payload remains attached to the linker, which can affect its electrical charge, hydrophobicity, or hydrophilicity. This may influence the payload’s ability to cross the cell membrane. Moreover, the linker itself can be expelled from the cell by efflux pumps, leading to the removal of the linker–payload complex. This can decrease the intracellular concentration of the payload and potentially contribute to drug resistance [27].

#### 2.1.4. Conjugation

Other than the structural details, conjugation is a crucial component for making an ADC more therapeutically effective. Most ADCs have traditionally been constructed using cysteine–maleimide alkylation or, less commonly, lysine–amide coupling. To lessen the chances of heterogeneity, different conjugation techniques are being used to improve payload delivery. The prime objective is to make homogenous conjugations which produce ADCs which are predictive in terms of their DAR. A few of the novel techniques of conjugation used are full alkylation of interchain disulfides used in T-DXd and sacituzumab govitecan, THIOMAB [28], incorporation of non-naturally occurring reactive amino acids [29,30], cysteine re-bridging [31,32], Fc-affinity tags [33], and site-specific conjugation using various enzymes (such as engineered glycosidases [34,35], transglutaminases [36,37], formyl glycine-generating enzymes [38,39], Fc-affinity peptides [40] (AJICAP-M) and sortases [41,42].

### 2.2. Mechanism of Action

**Figure 2:**
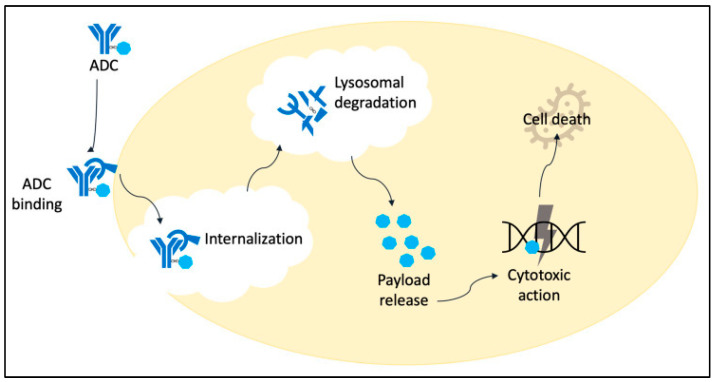
Mechanism of action of ADCs: Binding to the target, followed by internalization and release of cytotoxic payload, leading to cell death [44].

When the antibody–drug conjugate (ADC) binds to its target using the fragment antigen binding (Fab) region, the entire ADC–antigen complex is internalized into the cell through receptor-mediated endocytosis [43]. The payload is then released within an endosome or lysosome, contingent on the linker type. Eventually, the drug conjugate makes its way to the nucleus, where it implements its cytotoxic action on DNA, RNA, or microtubules, resulting in cell death, as demonstrated in Figure 2 [44].

## 3. ADCs in Gynecological Malignancies

The objective of ADCs is to enhance effectiveness while reducing the overall toxicity by delivering targeted cytotoxic therapy specifically to cancer cells. However, there is a possibility of on-target, off-tumor toxicities occurring when ADCs bind to non-cancer cells that also express the target antigen [45]. In the field of gynecological tumors, there are currently three ADCs that have been approved by the FDA, namely tisotumab vedotin, mirvetuximab soravtansine, and Trastuzumab Deruxtecan (T-DXd) [46,47].

### 3.1. ADCs in Ovarian Cancer

In the first-line treatment of advanced stage III/IV ovarian cancer, the current recommended approach is to combine optimal cytoreductive surgery with platinum-based chemotherapy. Additionally, the use of maintenance therapy after front-line therapy involving the antiangiogenic medication bevacizumab, as seen in the ICON7 [48] and GOG218 [49] trials, and/or poly (ADP-ribose) polymerase (PARP) inhibitors, as observed in trial SOLO 1, particularly in patients with BRCA mutations, has shown notable improvements in patient outcomes (HR 0.66; 95% CI 0.50 to 0.87) based on a network meta-analysis [50]. Although patients initially exhibit a high response rate, eventually 80% of them will encounter disease recurrence and a gradual development of resistance to chemotherapy [51]. In particular, when platinum resistance is present, there are few treatment options available, and the outlook is not favorable. Conventional treatments currently in use have low rates of response (15–20%) and a limited progression-free survival (PFS) of 3–4 months, with an overall survival (OS) of only 12 months [52]. In the context of recurrent disease, treatment options are often restricted due to the residual toxicity from previous therapies. The use of ADCs presents a valuable opportunity to enhance the effectiveness of chemotherapy while reducing systemic toxicities.

#### 3.1.1. Folate Receptor ADCs

Mirvetuximab soravtansine (MIRV) is an antibody–drug conjugate (ADC) that consists of a monoclonal antibody targeting the antifolate receptor α (FRα), a cleavable linker, and a potent antimitotic agent, DM4, which specifically targets tubulin [53]. The FRα receptor is a cell membrane protein responsible for binding and transporting folate into cells and is found in higher concentrations in epithelial tumors, especially high-grade serous ovarian and serous endometrial cancers, compared to its limited presence in normal adult tissues [54,55]. DM4 is electrically neutral and lipophilic, allowing it to penetrate cell membranes and generate the “bystander effect [56]”. In a phase I trial, the recommended dose of MIRV for solid tumors, including previously treated epithelial ovarian cancer (EOC), was established at 6 mg/kg every 3 weeks, with initial signs of activity observed [57]. Dose-limiting toxicities included grade 3 hypophosphatemia and grade 3 ocular toxicity, such as punctate keratitis [57]. A follow-up study on an expansion cohort of 46 patients with platinum-resistant EOC and FRα positivity, assessed through immunohistochemistry, revealed an overall response rate of 26%, with one complete response and 11 partial responses. The median progression-free survival (mPFS) was 4.8 months, and the median duration of response (DOR) was 19.1 weeks [58]. A phase Ib study confirmed the correlation between FRα expression levels and MIRV efficacy, with no objective response in patients with low FRα expression and an mPFS of 2.8 months [59]. However, the phase III FORWARD I trial, which compared MIRV with standard chemotherapy in platinum-resistant ovarian cancer, did not achieve its primary endpoint of progression-free survival (HR 0.98; 95% CI 0.73 to 1.31) [60]. In a subset of patients with high FRα expression, MIRV demonstrated anti-tumor activity, but the results were not statistically significant. Despite this, the study suggested a favorable benefit/risk safety profile compared to standard chemotherapy [60]. Based on these findings, two subsequent studies, MIRASOL and SORAYA, were initiated to evaluate the efficacy of MIRV in patients with high FRα expression. The main objective of the SORAYA study was to determine the confirmed objective response rate, as assessed by the investigator [61]. One hundred and six patients were included in the study, with one hundred and five being evaluated for effectiveness. All patients had previously received bevacizumab, with 51% having undergone three previous lines of therapy and 48% having received a prior poly ADP-ribose polymerase inhibitor. The median follow-up period was 13.4 months. The overall response rate was determined to be 32.4% (95% CI 23.6 to 42.2), with five complete responses and 29 partial responses. The median duration of response was 6.9 months (95% CI 5.6 to 9.7). Among patients with one to two prior treatments, the determined by the investigator was 35.3% (95% CI 22.4 to 49.9), while in patients with three prior treatments, it was lower, at 30.2% (95% CI 18.3 to 44.3). The overall response rate determined by the investigator was 38.0% (95% CI 24.7 to 52.8) in patients with prior exposure to a poly ADP-ribose polymerase inhibitor and 27.5% (95% CI 15.9 to 41.7) in those without such exposure. The most common treatment-related adverse events, both overall and of grade 3–4 severity, were blurred vision (41% and 6%), keratopathy (29% and 9%), and nausea (29% and 0%). These adverse events led to dose delays, reductions, and discontinuations in 33%, 20%, and 9% of patients, respectively. Ocular toxicity with MIRV was off target, as there are no folate receptor alpha receptors on the cornea.

The MIRASOL investigators conducted a phase III clinical trial to assess the efficacy and safety of mirvetuximab soravtansine compared to chemotherapy in patients with platinum-resistant, high-grade serous ovarian cancer [62]. Participants in this study had previously received 1 to 3 lines of treatment and exhibited high FRα tumor expression. A total of 453 participants were randomly assigned to receive either mirvetuximab soravtansine or chemotherapy, with 227 in the MIRV group and 226 in the chemotherapy group. For those on mirvetuximab soravtansine, the median progression-free survival (PFS) was 5.62 months (95% CI 4.34 to 5.95), compared to 3.98 months (95% CI 2.86 to 4.47) for those on chemotherapy, showing an improvement with MIRV. The overall response rate was 42.3% for the mirvetuximab soravtansine group versus 15.9% for the chemotherapy group. Overall survival (OS) was significantly longer with MIRV, with a median of 16.46 months compared to 12.75 months for chemotherapy (HR 0.67; 95% CI 0.50 to 0.89). Grade 3 or higher adverse events occurred less frequently with mirvetuximab soravtansine at 41.7%, compared to 54.1% for chemotherapy. The most common adverse effects were mild gastrointestinal, neurosensory, and reversible ocular events. Serious adverse events of any grade (23.9% vs. 32.9%) and discontinuation events (9.2% vs. 15.9%) were also lower with mirvetuximab soravtansine.

The ocular side effects of MIRV are not related to folate receptor targeting, as there are no folate receptor alpha receptors on the cornea. Damage to the cornea starts at the outer edges after mirvetuximab soravtansine travels to the cornea through the limbal region, where stem cells that accumulate DM4 are located [55]. These damaged stem cells move inward and cause the formation of small cysts in the cornea. The use of ocular steroids can slow down the growth of these damaged stem cells at the outer edges, reducing their sensitivity to the harmful effects of DM4. The cornea can regenerate new cells within seven to 10 days, so ocular side effects usually resolve within a week.

Other studies with mirvetuximab, such as the PICCOLO study, are currently exploring the use of mirvetuximab soravtansine as a stand-alone treatment for patients with ovarian cancer that is sensitive to platinum [56]. In the GLORIOSA study, mirvetuximab soravtansine is being compared to bevacizumab alone, as well as a combination of mirvetuximab soravtansine and bevacizumab, for maintenance therapy after a positive response to platinum in ovarian cancer that is sensitive to platinum [57]. Additionally, the combination of mirvetuximab soravtansine and carboplatin is being investigated as a second-line treatment option for patients with epithelial ovarian cancer that are sensitive to platinum [58,59]. In one ongoing trial, MIRV is being studied as part of a front-line neoadjuvant therapy in combination with carboplatin in advanced ovarian cancer [61].

Luveltamab tazevibulin (STRO-002) is an ADC that also specifically targets the folate receptor alpha (FRα) in tumor cells. It is composed of the FRα-binding antibody SP8166, a cleavable protease linker, and a hemiasterlin-derivative payload called SC209 [63]. The ADC has a cathepsin-sensitive linker that, when cleaved in the tumor microenvironment or upon internalization into tumor cells, allows for a targeted delivery and cytotoxic effect in tumor cells. We expect that this antibody–drug conjugate will be more stable and have less toxicity, as it is more stable in blood, and SC209 demonstrates rapid clearance. In addition, SC209 is less likely to be pumped out of cells by the efflux pump P-glycoprotein, making STRO-002 a more potent treatment option for ovarian cancers that are resistant to other therapies like platinum or PARP inhibitors [63]. Another feature in favor of STRO-002 is its ability to produce bystander killing of neighboring tumor cells that do not express FRα, further enhancing its effectiveness in tumors with heterogeneous or low FRα expression. The hemiasterlin-derivative payload of STRO-002 not only inhibits tubulin, but also stimulates an immunogenic response upon cell death [63].

Preliminary data from a phase I dose escalation study in advanced ovarian cancer patients showed that a higher dose of STRO-002 resulted in a higher overall response rate (43.8%) compared to a lower dose with an overall response rate of 31.3%. The safety profile was also acceptable with most treatment-related adverse events being grades 1 or 2 and no ocular toxicity reported [64]. Most of the studies are ongoing and results are expected to be published in coming years. A phase I STRO-002-GM2 study (NCT05200364) is aimed at evaluating the combination of STRO-002 and bevacizumab in patients with advanced platinum-resistant ovarian cancer. The main goal of the STRO-002-GM2 study is to determine the recommended phase 2 dose (RP2D) of the STRO-002/bevacizumab combination and assess its safety. Secondary and exploratory objectives include investigating the pharmacokinetics (PK) and preliminary anti-tumor activity of the combination [65]. REFRaME-O1 (NCT05870748) is a two-part phase II trial evaluating the efficacy and safety of luveltamab tazevibulin in patients with relapsed platinum-resistant epithelial ovarian cancer expressing folate receptor alpha. In Part 1, it involves two dosing cohorts (Cohort A and Cohort B) with a 1:1 randomization, which aims to optimize the dosing in Part 1, and then Part 2 will further evaluate the efficacy and safety of the selected dosing regimen [66].

Another ADC targeting FRα is MORAb-202 (farletuzumab ecteribulin), which consists of a farletuzumab antibody aimed at FRα, combined with the cytotoxic agent eribulin mesylate through a cleavable linker. In a phase I dose-escalation study involving FRα-positive solid tumors, MORAb-202 achieved a disease control rate of 75%, with one complete response and two partial responses among nine ovarian cancer patients [67]. Additionally, a phase I/II trial is investigating MORAb-202 in various tumor types, including endometrial and platinum-resistant ovarian cancer. Eligible endometrial cancer patients must have experienced relapse or failure following at least one prior platinum-based chemotherapy or one immunotherapy regimen [67] (NCT04300556). Currently, MORAb-202 is undergoing a phase II trial to compare its efficacy with the investigator’s choice of chemotherapy in patients with platinum-resistant high-grade serous ovarian, peritoneal, or fallopian tube cancer [68] (NCT05613088).

Another upcoming folate receptor alpha (FRα) ADC is AZD5335; its preliminary data were recently presented [69,70]. AZD5335 is a new ADC with an antibody portion targeting folate receptor alpha (FRα) and a conjugated topoisomerase 1 inhibitor (TOP1i) as a payload. It was reported that a single dose of AZD5335 at 2.5 mg/kg was sufficient to provide a solid and consistent anti-tumor response in FRα-expressing ovarian cancer cell line xenografts (CDX) with a tumor growth inhibition (TGI) of 75–94% and median best tumor volume reduction of >30% in 14/17 (82%) ovarian cancer patient-derived xenografts (PDX). An ongoing phase I/IIa study for AZD5335 as monotherapy and in combination with anti-cancer agents in participants with solid tumors (FONTANA) is recruiting patients. It will assess safety and tolerability along with response rate, duration of response, disease control rate, and progression-free and overall survival [71] (NCT05797168).

#### 3.1.2. Other ADCs

##### MUC 16

Another drug, DMUC4064A, is a monoclonal antibody that targets MUC16, a protein overexpressed in most epithelial ovarian cancers. The antibody is conjugated to monomethyl auristatin E, a microtubule-disrupting agent. A phase I/II study was conducted to evaluate the safety, tolerability, pharmacokinetics, and preliminary activity of DMUC4064A in patients with platinum-resistant ovarian cancer (OC). A total of 65 patients with platinum-resistant OC were enrolled in the study. They received DMUC4064A once every 3 weeks in dose escalation cohorts. The patients received a median of 5 cycles of DMUC4064A. The maximum tolerated dose was not reached, and the recommended phase II dose (RP2D) was determined on the overall tolerability profile. The most common adverse events reported by patients included fatigue, nausea, abdominal pain, constipation, blurred vision, diarrhea, and anemia. The study did not report on the preliminary activity or efficacy of DMUC4064A in treating platinum-resistant OC. In conclusion, the study found that DMUC4064A was generally well-tolerated in patients with platinum-resistant OC, with the RP2D determined. Further studies are needed to evaluate the efficacy of DMUC4064A in treating platinum-resistant OC [72].

##### Mesothelin

Mesothelin is a cell membrane glycoprotein primarily present in the mesothelial cells lining the pleura, pericardium, and peritoneum. While its expression in normal tissues is limited, it is significantly overexpressed in various cancers, including up to 70% of ovarian cancer cases [73]. Its involvement in cell adhesion and metastasis makes mesothelin an appealing target for cancer-specific therapies [74]. Several anti-mesothelin antibody–drug conjugates (ADCs) are being explored, with anetumab ravtansine (BAY 94-9343) being a notable example. This ADC is composed of a fully humanized monoclonal antibody directed at mesothelin, a disulfide linker, and the cytotoxic agent DM4, a tubulin inhibitor [74]. In a study involving 65 patients with platinum-resistant epithelial ovarian cancer, anetumab ravtansine was administered with pegylated liposomal doxorubicin intravenously every three weeks. During the dose escalation phase, nine patients received two different doses of anetumab ravtansine without experiencing any dose-limiting toxicities. In the dose expansion phase, 56 patients were treated at the maximum tolerated dose. The most frequent side effects included nausea (47.7%), decreased appetite (43.1%), fatigue (38.5%), diarrhea (32.3%), and corneal disorder (29.2%). The overall objective response rate was 27.7%, with one complete response and 17 partial responses. The median duration of response was 7.6 months and the median progression-free survival was 5.0 months. Among patients with high mesothelin expression and three or fewer previous systemic therapies, the objective response rate was 42.1%, with a median response duration of 8.3 months and median progression-free survival of 8.5 months [75].

##### CDH6

Cadherin-6 (CDH6) is a transmembrane protein expressed in many cancers, including epithelial ovarian cancers [76]. A novel ADC, raludotatug deruxtecan (R-DXd) is a CDH6 protein-targeting antibody–drug conjugate [77]. A first-in-human phase I study recruited 42 patients with ovarian cancer; all were platinum resistant, 29 (69%) had received prior bevacizumab, and 26 (62%) had received prior PARP inhibitors. As per the latest update, half of the patients were still receiving the treatment. Treatment-emergent adverse events (TEAEs) were experienced by 37 patients (88%), and grade ≥ 3 TEAEs were observed in 21 (50%). The most common all-grade TEAEs were nausea (55%), fatigue (40%), vomiting (38%), and diarrhea (33%). Adverse effects led to R-DXd discontinuation in 14% of patients. In regards to efficacy, the overall response rate was 38%, with 1 CR, and 11 out of 21 patients were showing down-trending Ca-125 [78] (NCT04707248).

### 3.2. ADCs in Cervical Cancer

Cervical cancer has a 5-year survival rate of 67% [2,79]. Currently, the recommended initial treatment for patients with recurrent or metastatic cervical cancer is a combination therapy consisting of pembrolizumab, bevacizumab, and a chemotherapy doublet of paclitaxel and platinum, based on the PDL1 status [80]. In the second-line setting, there are limited options available. Available cytotoxic agents such gemcitabine, irinotecan, and pemetrexed have low levels of efficacy. Patients who are naïve to immune checkpoint inhibitor could be considered for pembrolizumab or cemiplimab in second-line treatment [80].

#### Tisotumab Vedotin

Many solid tumors, including cervical cancers, express high levels of Tissue Factor (TF), which can promote tumor growth, metastasis, and angiogenesis [46]. Tisotumab vedotin (TV) is an ADC targeting TF. GOG-3023/ENGOT-cx6/innovaTV204 evaluated the effectiveness and safety of TV in patients with previously treated recurrent or metastatic cervical cancer [46]. The phase 2 trial which led to its FDA approval enrolled 102 patients who had experienced disease progression after receiving doublet chemotherapy with or without bevacizumab. Eligible patients had received a maximum of two prior systemic treatment regimens for recurrent or metastatic cancer. During the trial, patients received TV intravenously at a dose of 2 mg/kg every 3 weeks until disease progression or unacceptable toxicity. The primary endpoint of the trial was the objective response rate based on the RECIST criteria. Secondary endpoints included safety analysis. The confirmed objective response rate was 24%, with 7% of patients achieving a complete response (CR) and 17% experiencing a partial response (PR). The median duration of response was 8.3 months. An exploratory analysis showed that patients responded to TV, regardless of the level of membrane tissue factor expression [46].

In InnovaTV 205/ENGOT-cx8/GOG-3024, tisotumab vedotin (TV) was found to be safe without any drug-related toxicities when combined with carboplatin, bevacizumab, and pembrolizumab. For TV given as first-line treatment, the objective response rate was 54.5% with carboplatin, 40.6% with pembrolizumab, and 35.3% with 2nd-line/3rd-line TV + pembrolizumab (arm F). The median duration of response was 8.6 months, not reached, and 14.1 months in arms D, E, and F, respectively. The grade ≥ 3 adverse events (≥15%) observed were anemia, diarrhea, nausea, and thrombocytopenia in arm D and anemia in arm F (none ≥ 15% in arm E).

Further, a recently published phase III randomized trial, innovaTV 301/ENGOT-cx12/GOG-3057, reported that when used as a second- or third-line treatment for patients with recurrent or metastatic cervical cancer that has progressed on doublet chemotherapy, there was a 30% decrease in the risk of death compared to the investigators’ choice of chemotherapy [81]. The findings revealed that after one year of follow up, the overall survival (OS) was 48.7% when using TV, compared to 35.3% with chemotherapy (HR, 0.70; 95% CI 0.54 to 0.89). In terms of progression-free survival (PFS), it was 30.4% with TV versus 18.9% with chemotherapy (HR, 0.67; 95% CI, 0.54 to 0.82). The disease control rate was 75.9 with TV compared to 58.2% with chemotherapy. The median duration of response (DOR) was 5.3 months for TV and 5.7 months for chemotherapy. The most common adverse events observed with TV included conjunctivitis, peripheral sensory neuropathy, alopecia, epistaxis, decreased appetite, diarrhea, and keratitis. Overall, the rates of these adverse events were significantly higher with TV compared to chemotherapy. Grade 1 to 3 adverse events were observed with TV, but no grade 4 adverse events were reported. Ocular events, peripheral neuropathy, and bleeding were the most common adverse events associated with TV. One patient with another tumor type treated with tisotumab vedotin at the recommended dose developed Guillain–Barre syndrome [82].

### 3.3. ADCs in Endometrial Cancer

In Canada, endometrial cancer is the second most prevalent and second most fatal gynecologic cancer [2]. The Cancer Genome Atlas has identified four molecular subtypes that have an impact on prognosis, leading to the recommendation of subtype-specific treatment considerations [83]. For previously treated cases with deficient mismatch repair/high microsatellite instability, pembrolizumab and dostarlimab have been approved by the FDA, while pembrolizumab/lenvatinib are approved for previously treated cases as a second-line treatment in cases with proficient mismatch repair/microsatellite stability [84]. However recently published data from RUBY and NRG-GY018 trials immunochemotherapy have shown promising results in dMMR, as well as pMMR subgroups, respectively [85,86]. Within this focused molecular landscape, there is ongoing research on ADCs.

Currently, there is a growing body of positive data on three sets of ADCs. Firstly, there are promising data on ADCs targeting HER2, particularly trastuzumab deruxtecan (T-DXd), which has shown positive results in terms of response rates, particularly in cases of serous subtype of endometrial cancer with HER2 expression. The activity of T-DXd will be discussed further in Section 3.4 below. Secondly, there are encouraging data on ADCs targeting Trop-2, specifically sacituzumab govitecan, which has shown positive results in terms of response and survival in endometrioid and serous subtypes of endometrial cancer where Trop2 is more commonly expressed [87].

Trop-2 is a tumor-associated calcium signal transducer found to be highly expressed in various types of endometrial cancer (EC), including grade 3 endometrioid adenocarcinoma (96%) and uterine serous carcinoma (65%). Its overexpression is associated with a poorer prognosis and increased likelihood of disease recurrence. Sacituzumab govitecan (IMMU-132) is an ADC that consists of a humanized anti-Trop-2 antibody linked to the active form of irinotecan, a topoisomerase-I inhibitor. In a phase 2 study in endometrial cancer, 21 patients were enrolled, including 48% with uterine serous carcinoma, 33% with endometrioid adenocarcinoma, 14% with carcinosarcoma, and one patient with mixed serous and clear cell histology. All patients had received at least one prior line of chemotherapy, with a median of three lines and a range of one to six. Among the 20 patients evaluated for response, 35% achieved an objective response. Eighteen patients were evaluated for durable disease control, with 39% achieving it. The median follow-up duration was 15.6 months. The median overall survival was 22.5 months and the median progression-free survival was 5.7 months. The treatment was well-tolerated, with no new or unexpected safety concerns reported [87].

Other Trop-2-targeting ADCs are under investigation. Datopotamab deruxtecan is an ADC made up of a highly effective topoisomerase I inhibitor payload chemically attached to a humanized anti-Trop-2 IgG1 monoclonal antibody using a tetra peptide-based cleavable linker that is stable and tumor-selective [88]. Dato-DXd is undergoing evaluation in a pan-tumor phase 2 trial which includes an endometrial cancer cohort (TROPION-PanTumor03, NCT05489211).

Another novel ADC targeting Trop-2 is SKB264. It utilizes the same monoclonal antibody as IMMU-132 and contains 7–8 molecules of a new toxic payload linked through disulfide bonds. The toxic payload, KL610023 (T030), is a belotecan derivative that inhibits topoisomerase I [73]. SKB264 has a longer half-life compared to IMMU-132 and exhibits stronger targeting and bystander toxicity [73]. Currently, there are ongoing phase I/II studies, such as (NCT04152499) and (NCT05642780), that are investigating dose escalation and combination approaches with immunotherapy [75,89].

Endometrial tumors also commonly exhibit an overexpression of FRα receptors, similar to ovarian cancer, with approximately 64% of endometrial tumors testing positive for FRα [74]. However, the clinical effectiveness of the anti-FRα ADC mirvetuximab soravtansine has not been as clear, despite promising preclinical data [90]. In a study examining multiple solid tumors, a positive response was observed in 2 out of 11 (18.2%) endometrial tumors when administered at a dose of 5 mg/kg once every 3 weeks [91]. Currently, a phase II trial is underway to assess the combination of mirvetuximab soravtansine and Pembrolizumab in microsatellite-stable endometrial cancer [92] (NCT03835819).

### 3.4. HER2 ADCs in Gynecologic Cancers

#### 3.4.1. Trastuzumab Deruxtecan (T-DXd)

Trastuzumab deruxtecan is a HER2-specific antibody drug conjugate currently approved for the treatment of HER2-low metastatic breast cancer. The active payload, deruxtecan, is a potent DNA topoisomerase I inhibitor which is more potent than the irinotecan derivative SN-38. The interim findings from the DESTINY-PanTumor-02 study demonstrated activity of the ADC trastuzumab deruxtecan (T-DXd) in various tumor cohorts, including ovarian, endometrial, and cervical cancers. The study included 267 patients across six specific tumor cohorts, with an overall response rate of 37.1%. The median duration of response (DOR) was 11.3 months, median progression-free survival (PFS) was 6.9 months (95% CI, 5.6 to 8.0), and median overall survival (OS) was 13.4 months (95% CI, 11.9 to 15.5). In patients with central HER2 immunohistochemistry (IHC) 3+ expression, the objective response rate was 61.3%, with a median duration of response of 22.1 months, median PFS of 11.9 months, and median OS of 21.1 months. Grade ≥ 3 drug-related adverse events occurred in 40.8% of patients, with 10.5% experiencing drug-related interstitial lung disease (ILD) and three deaths related to ILD. The response rates were particularly high in patients with cervical, endometrial, and ovarian cancer, at 50.0%, 57.5%, and 45.0% respectively. In all cohorts, higher response rates were demonstrated in patients with HER2 3+ expression compared to those with HER2 2+ expression. This trial suggested T-DXd as a reasonable treatment option for all cancer types with HER2 overexpression. However, caution should be exercised due to the occurrence of ILD-related adverse events [93]. Recently, FDA granted accelerated approval to fam-trastuzumab deruxtecan for unresectable or metastatic HER2-positive solid tumors, agnostic to tumor type [94].

The results of the STATICE, a phase 2 trial, showed promising outcomes for T-DXd in the treatment of uterine carcinosarcoma with HER2 positivity. A total of 33 patients received T-DXd out of 84 screened patients. The objective response rate, as evaluated by central review, was 54.5% in the HER2-high group and 70.0% in the HER2-low group. The median progression-free survival (PFS) and overall survival (OS) in the HER2-high group were 6.2 and 13.3 months, respectively, while in the HER2-low group, the median PFS was 6.7 months and the OS was not reached. Grade ≥ 3 adverse events were observed in 20 patients (61%). Pneumonitis/interstitial lung disease of grades 1–2 and grade 3 occurred in eight (24%) and one (3%) patient(s), respectively [95].

#### 3.4.2. Trastuzumab Duocarmazine

Trastuzumab duocarmazine (SYD985) is a combination of the monoclonal antibody trastuzumab, which targets HER2, and a duocarmycin derivative. The duocarmycin payload is attached to the antibody through a cleavable linker and includes a prodrug called seco-duocarmycin-hydroxybenzamide-azaindole (seco-DUBA) [96]. This payload acts by alkylating DNA, causing DNA damage and ultimately leading to cell death [96]. During a phase I clinical trial that aimed to expand the dose of trastuzumab duocarmazine in patients with HER2-positive breast, gastric, urothelial, or endometrial cancer, a total of 146 patients were included. Among these patients, 14 had endometrial cancer and received a dosage of 1.2 mg/kg of trastuzumab duocarmazine every 3 weeks. Out of the 14 patients with endometrial cancer, five (39%, 95% CI 13.9 to 68.4) showed partial disease responses [97]. The results of another study [98] (NCT04205630), which is a phase II trial, are currently being awaited. This study is an open-label, single-arm trial that includes patients with recurrent, advanced, or metastatic endometrial carcinoma expressing HER2. HER2 expression is determined by a score of 1+, 2+, or 3+ on immunohistochemistry or positive results on in situ hybridization. Patients eligible for this study must have experienced progression after first-line platinum-based chemotherapy, while those who have undergone two or more lines of chemotherapy for advanced or metastatic disease are not eligible. Eligible patients will be administered SYD985 until disease progression or unacceptable toxicity occurs. The results of this study are currently pending. Another phase 1 study involving 32 patients has been completed and is currently awaiting results. This study is a two-part phase 1 trial that aims to assess the safety, pharmacokinetics, and efficacy of the ADC SYD985 when combined with Niraparib in patients with locally advanced or metastatic solid tumors expressing HER2 [99].

### 3.5. Miscellaneous ADCs

Other than folate receptor alpha (FRα), NaPi2B, a protein involved in sodium-dependent phosphate transport, is found in approximately two thirds of high-grade serous ovarian cancer patients. Upifitamab rilsodotin (UpRi) is an ADC that specifically targets NaPi2B. However, the phase 1b/2 UPLIFT trial (NCT03319628) did not meet its primary endpoint of achieving a satisfactory objective response rate as assessed by investigators. Out of 141 NaPi2B-positive patients receiving UpRi, only 22 showed a response, resulting in an investigator-assessed objective response rate of 15.6% (95% CI, 10.0 to 22.7) [100,101,102]. Further investigations of UpRi are being conducted in combination with carboplatin in high-grade serous ovarian cancer patients in the phase 1 UPGRADE trial (NCT04907968), as well as in a phase 3 randomized UP-NEXT trial (NCT05329545) where it is being evaluated as a maintenance therapy compared to a placebo in patients with platinum-sensitive recurrent ovarian cancer. However, the FDA has placed a hold on patient enrollment for both the UP-NEXT and UPGRADE-A trials that are assessing UpRi [103], due to the higher-than-expected rates of bleeding observed.

### 3.6. Next Generation ADCs

Recently, newer techniques have been incorporated and next-generation ADCs have been created. Keeping tumor heterogeneity in consideration, bispecific antibodies have emerged as a way to enable simultaneous binding to two distinct target molecules or cells [104]. A few examples of biparatropic ADCs which target different epitopes of Her2 are under investigation, a notable example being MEDI4276, containing 4 antigen binding sites and targeting 2 epitopes [105,106]. Another anti-Her2 targeting biparatropic ADC is Zanidatamab Zovodotin [107]. ADCs often target known receptors which are not only expressed in tumor cells but also normal tissues. To overcome this cross-reactivity, probody–drug conjugates (PDCs) are under development. Praluzatamab Ravtansine (CX-2009) is a conditionally activated PDC, a CD166-targeting ADC that has recently been explored in epithelial ovarian epithelial cancer [108]. A few more examples of immune-stimulating ADCs, which carry immune stimulators as payloads, are under development. Degrader–Antibody Conjugates (DACs) are composed of an antibody that targets a specific protein on the surface of cancer cells and a small molecule degrader that binds to the targeted protein and induces its degradation; these have also been around and hold promise. Another example is ADCs which can deliver dual chemotherapeutic agents. We have summarized the completed as well as ongoing trials of selected ADCs in Table 1.

## 4. Beyond ADCs in Gynecological Cancers

At the moment there is no clear consensus how to treat recurrent/refractory gynecological cancers. Chemotherapy at this stage is usually not effective and can lead to unnecessary toxicities. Depending upon the molecular profile, certain targeted agents can be used. For example, Larotrectinib or Entrectinib can be used in NTRK fusion-positive tumors, and Selpercatinib is also an option for RET gene fusion-positive tumors.

## 5. Conclusions

ADCs, a novel and innovative approach, are being incorporated in the management of gynecologic cancers. Ongoing trials aim to identify effective agents for treating gynecologic cancers, particularly in cases where the cancer has spread or recurred and treatment options are limited. In general, ADCs have been shown to be more effective than traditional chemotherapy regimens, with less toxicity, especially in resistant cases. However, it is important to be cautious of the fact that ADCs can still cause specific side effects, such as ocular toxicity and neuropathy. Therefore, it is crucial to take preventive measures, closely monitor patients, and promptly address any issues that arise during treatment. While most agents have been studied as standalone treatments, researchers are considering combining them with other therapies to potentially achieve longer-lasting responses, although this may increase the risk of cumulative toxicity. ADCs have emerged as promising therapeutic options for gynecologic cancers, and ongoing and future research may help improve patient outcomes while minimizing treatment-related side effects.

## Figures and Tables

**Table 1:** Summary of selected ongoing clinical trials of ADCs in gynecological cancers.

ADC	Target	Payload	Mechanism of Action	Gynecologic Cancer Type	Phase	Clinical Trials Identifiers	Key Arms
Mirvetuximab Soravtansine (IMGN853)	FRα	DM4	Inhibition of tubulin polymerization	Ovarian	III	MIRASOL NCT04209855	MIRV vs. Investigator choice chemotherapy
					II	MIROVA (NCT04274426)	Carboplatin + MIRV -> MIRV maintenance vs. platinum-based chemotherapy
					II	PICCOLO (NCT05041257)	MIRV monotherapy
					III	GLORIOSA (NCT05445778)	MIRV + bevacizumab vs. bevacizumab monotherapy
Luveltamab tazide (STRO-002)	FRα	SC209	Inhibition of tubulin polymerization	Ovarian, Fallopian Tube, Primary Peritoneal, Endometrial	I/II	NCT03748186 NCT05870748	STRO-002 at escalating doses
					I	NCT05200364	STRO-002 at escalating doses + Bevacizumab
Farletuzumab Ecteribulinm (MORAb-202)	FRα	Eribulin	Inhibition of microtubules	Ovarian, Fallopian Tube, Primary Peritonel, Endometrial	I	NCT03386942	MORAb-202 at escalating doses
					I/II	NCT04300556	MORAb-202 at escalating doses
					II	NCT05613088	MORAb-202 at 2 different doses vs. Chemotherapy
Trastuzumab deruxtecan (DS-8201a)	HER2	Deruxtecan	Inhibition of topoisomerase I	Endometrial, Ovarian, Cervical	II	DESTINY-PanTumor02 NCT04482309	DS-8201a monotherapy
					I	NCT04585958	Trastuzumab Deruxtecan + olaparib
DB-1303	HER2	P1003	Inhibition of topoisomerase I	Endometrial	I/IIA	NCT05150691	DB-1303 dose monotherapy
Trastuzumab Duocarmazine (SYD985)	HER2	Duocarmycin	DNA alkylation	Endometrial, Ovarian	II	NCT04205630	SYD985 monotherapy
					I	NCT04235101	SYD985 + Niraparib at various doses
Tisotumab vedotin (HuMax-TF-ADC)	TF	MMAE (monomethyl auristatin E)	Inhibition of tubulin polymerization	Cervical	III	NCT04697628 (innovaTV 301)	Tisotumab Vedotin vs. another chemotherapy regimen
DMUC4064A	MUC16	MMAE (monomethyl auristatin E)	Inhibition of tubulin polymerization	Ovarian	I	NCT02146313	DMUC5754A at escalating doses
Anetumab ravtansine (BAY94-9343)	Mesothelin	DM4	Inhibition of microtubule polymerization	Ovarian, Fallopian Tube, Primary Peritoneal	I	NCT02751918	BAY94-9343 + Pegylated Liposomal Doxorubicin
					I	NCT01439152	BAY94-9343 at escalating doses
					II	NCT03587311	BAY94-9343 + Bevacizumab vs. Paclitaxel + Bevacizumab
Upifitamab Rilsodotin (XMT-1536) Discontinued	NaPi2b	Auristatin derivative	Inhibition of tubulin polymerization	Ovarian, Fallopian Tube, Primary Peritoneal	Ib/II	UPLIFT NCT03319628	UpRi at escalating doses
					I	UPGRADE-A NCT04907968	UpRi at escalatig doses +Carboplatin
					III	UP-NEXT NCT05329545	UpRi vs. Placebo
Sacituzumab govitecan (IMMU-132)	TROP2	SN-38 (irinotecan metabolite)	Inhibition of topoisomerase I	Endometrial	II	NCT04251416	IMMU-132 monotherapy
					II	NCT03964727	IMMU-132 monotherapy
SKB264	TROP2	Proprietary belotecan derivative	Inhibition of topoisomerase I	Ovarian, Endometrial, Cervical	I/II	NCT04152499	SKB264 at escalating doses
					II	NCT05642780	SKB264 + Pembrolizumab
Raludotatug Deruxtecan (R-DXd; DS-6000)	CDH6	Deruxtecan	Inhibition of topoisomerase I	Ovarian Cancer	I	NCT04707248	R-DXd, DS-6000 monotherapy
